# The First Identification of *Trichinella britovi* in the Raccoon Dog (*Nyctereutes procyonoides)* in Romania

**DOI:** 10.3390/pathogens12091132

**Published:** 2023-09-05

**Authors:** Ana-Maria Marin, Dan-Cornel Popovici, Gheorghe Dărăbuș, Cătălin Marian, Diana Nițușcă, Narcisa Mederle

**Affiliations:** 1 Faculty of Veterinary Medicine, University of Life Sciences “King Michael I” from Timisoara, 300645 Timisoara, Romania; anamaria.marin@usvt.ro (A.-M.M.); gheorghe.darabus@fmvt.ro (G.D.); narcisamederle@usvt.ro (N.M.); 2 Forestry Faculty, Transilvania University Brasov, No. 1 Sirul Beethoven, 500123 Brasov, Romania; 3 Department of Biochemistry and Pharmacology, “Victor Babes” University of Medicine and Pharmacy from Timisoara, No. 2 Piaţa Eftimie Murgu, 300041 Timisoara, Romania; cmarian@umft.ro (C.M.); nitusca.diana@umft.ro (D.N.)

**Keywords:** *Trichinella* spp., Romania, raccoon dog

## Abstract

*Trichinella* spp. are nematodes distributed throughout the world that affect an impressive number of host animals (mammals, birds, and reptiles) involved in the evolution of two cycles, the domestic and the sylvatic. The raccoon dog (*Nyctereutes procyonoides*) is an omnivorous mammal with great ecological plasticity. The expansion of the raccoon dog in Europe is associated with the risk of the introduction and spread of different pathogens, especially zoonotic ones (*Trichinella, Echinococcus*). Currently, the raccoon dog’s range in Romania is limited to the Danube Delta area, the Lower Danube Meadow, and the Prut Meadow. The aim of this study was to examine the presence of *Trichinella* larvae isolated from the muscles of raccoon dog from six hunting funds of Giurgeni, Ialomița County, Romania. The muscle samples were examined via artificial digestion, and the obtained larvae were processed via multiplex PCR. The PCR-amplified ESV and ITS1 DNA fragments were then sequenced for species confirmation. The species *Trichinella britovi*, which is the most common species identified in wild carnivores in temperate zones, was confirmed. Although *T. britovi* has been reported in several host animals in Romania, this case report confirms its presence in the raccoon dog for the first time.

## 1. Introduction

A species considered non-native in Europe, the raccoon dog (*Nyctereutes procyonoides*, Gray 1834), has maintained a constant presence in the wild fauna of Romania, especially since the second half of the 20th century [1]. This species, originally from southeast Siberia (Amur and Ussuri river basin), arrived in Romania through natural expansion from the European area of Russia, where it was artificially introduced at the beginning of the 20th century. Some authors consider the subspecies *Nyctereutes procyonoides ussuriensis* to be that which was spread in eastern and northern Europe, with this species also currently expanding in central Europe [1].

A species with high ecological plasticity, the raccoon dog has an omnivorous diet, which is highly variable depending on the type of habitat used and the season, this being an extremely opportunistic animal that capitalizes on a varied range of foods (from fruits and plants to amphibians, reptiles, eggs, and chicks). The types of habitats used by this species are characterized by an excess of humidity, especially in areas of meadows with watercourses and rich vegetation and marshy areas that alternate between open spaces and flooded pastures [1].

Within its range, the raccoon dog competes directly with other canid species, especially with the fox (*Vulpes vulpes*) and the jackal (*Canis aureus*). However, it has exhibited real success in the process of invading and expanding its European range due to a combination of factors, among which we should mention its high reproductive capacity, high degree of adaptability to various habitat conditions (being the only species of this genus that can hibernate in winter), and remarkable dispersion capacity [1].

Almășan H., 1951, noted the presence of this species in Romania for the first time [2]. Currently, the range of the raccoon dog in Romania is limited to the area of the Danube Delta, the Lower Danube Meadow, and the Prut Meadow, this species being stable and observed within the range of 12 hunting funds from five counties.

This carnivore invaded Europe from the East, and its contribution to the epidemiology of parasitic diseases is evident in that this reservoir host represents a threat to the health of animals and humans [3,4,5,6].

The expansion of the raccoon dog in Europe is associated with the risk of the introduction and spread of different pathogens, especially zoonotic ones (*Trichinella, Echinococcus*). The species of the genus *Trichinella* are nematodes distributed throughout the world that affect an impressive number of host animals (mammals, birds, and reptiles) involved in the evolution of two cycles, the domestic and the sylvatic [7,8].

In Europe, the red fox (*Vulpes vulpes*) and the raccoon dog are considered the most important reservoir hosts [9]. Mustelids (badgers, beech marten, etc.) and other carnivores (bears, lynxes, wolves, etc.) can also represent sources of infection, but they only play a secondary role in the ecology of the sylvatic cycle [9]. Knowledge of the particularities of the sylvatic cycle is important for trichinellosis, in regard to which the double risk of *Trichinella* infection has been emphasized, whether through the consumption of undercooked game meat (especially wild boar) or the transmission of *Trichinella* strains from wildlife to domestic animals (pigs raised in yards) [7].

*Trichinella* spp. are the etiological agents of trichinellosis, which is a foodborne human disease caused by the consumption of raw or undercooked meat of animals infected with the larvae of these zoonotic nematodes [8]. The main reservoir hosts of these pathogens are carnivorous and omnivorous mammals, birds, and reptiles. These hosts are distributed throughout all continents except Antarctica [7].

Currently, 13 taxa are included in the Trichinella genus, namely, the encapsulated species *T. spiralis, T. nativa, T. britovi, T. murrelli, T. nelsoni, T. patagoniensis*, and *T. chanchalensis* and the *Trichinella* genotypes T6, T8, and T9, exclusively for mammals. The non-encapsulated species are *T. pseudospiralis, T. papuae*, and *T. zimbabwensis*, which infect mammals and birds or mammals and reptiles [7,8,9].

In Romania, trichinellosis is a zoonosis with a high level of infection. The improper consumption of pork or game (boar, bear) represents the major route of human infection [10,11].

In Romania, no case of infection of the raccoon dog with the larvae of *Trichinella* spp. has been reported thus far [10]. Although in Romania, there are no reports supporting the possible consumption of raccoon dog meat, it appears to be an invasive species and a suitable host for *Trichinella* species [12]. The purpose of this study was to provide the first report of *Trichinella* infection in a raccoon dog collected for hunting purposes in Romania. 

We also carried out species identification using mPCR and a comparison with other species deposited in the Genbank database.

## 2. Materials and Methods

For the detection of *Trichinella* infection in animals, direct trichinoscopy and artificial digestion are valuable methods for diagnosis [13]. For the detection of *Trichinella* infection in humans, serological tests for detecting *Trichinella*-specific antibodies are valuable methods [14,15]. For identifying the species or genotype of *Trichinella* larvae, the recommended methods are multiplex polymerase chain reaction (mPCR) or, in rare cases, other PCR-derived methods and DNA sequencing [16,17].

### 2.1. Case Report

#### 2.1.1. The Study Area

**Figure 1:**
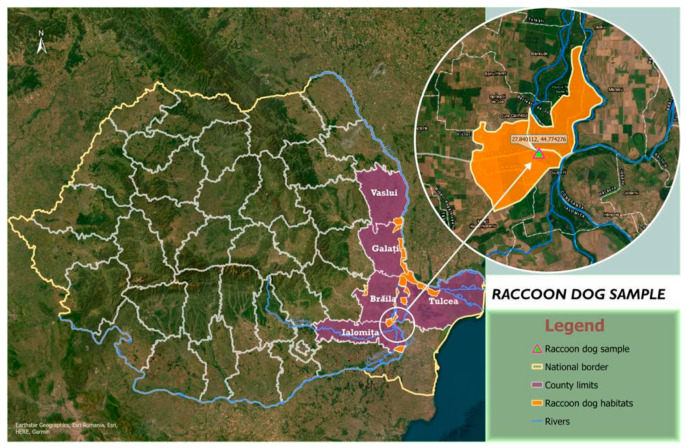
The map shows the study area from which the carcass of the raccoon dog was taken.

On 13 March 2023, a male raccoon dog was shot based on the annual harvest quota approved by the Minister of the Environment, Water and Forests, number 1571/2022. The hunting action through which this raccoon dog specimen was harvested was carried out in compliance with Law 407/2006 (on hunting and wildlife protection) [18]. This animal was harvested by hunters on the number six Giurgeni hunting grounds, Ialomița County, and transported under legal conditions to Faculty of Veterinary Medicine/ University of Life Sciences from Timisoara. The animal was examined in the Parasitic Diseases Clinic of the Faculty of Veterinary Medicine Timisoara/ULST (Figure 1).

**Figure 2:**
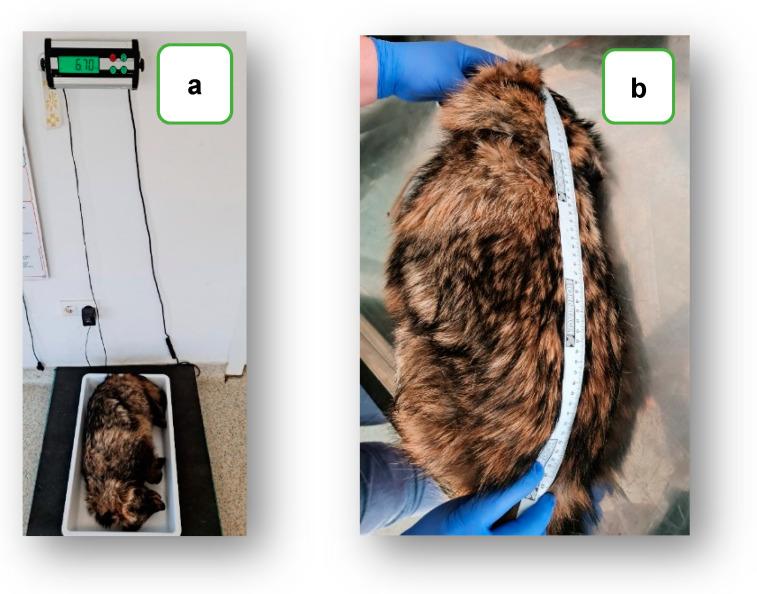

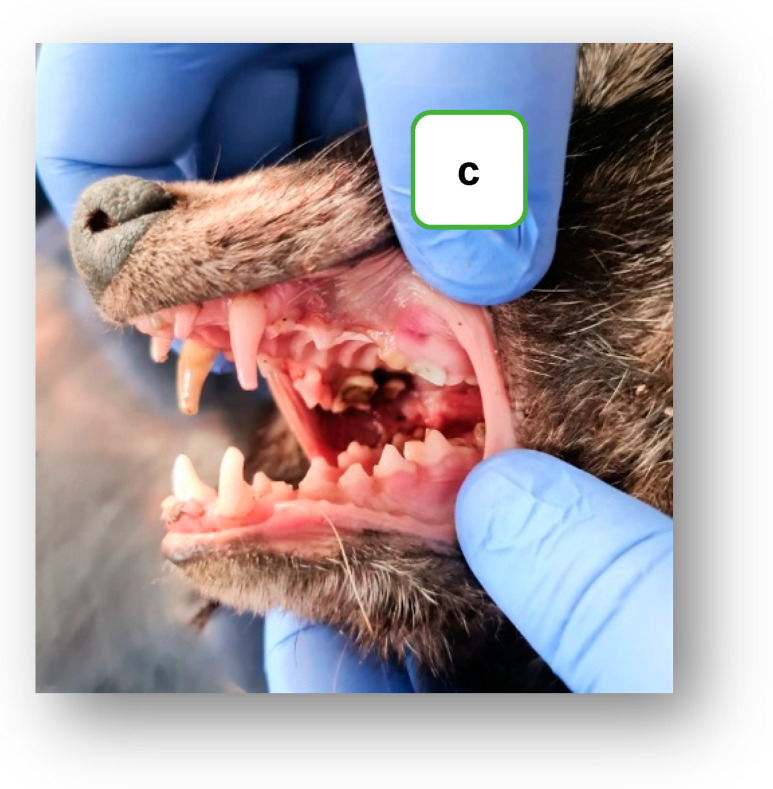
Evaluation of the main morphological parameters: weight—(**a**), length—(**b**), and age estimation based on the degree of dentition wear—(**c**).

The raccoon dog specimen weighed 6.7 kg, with a length of 57 cm (from the tip of the nose to the base of the tail) and an age over 3 years, which was judging by the degree of dentition wear (Figure 2a–c).

#### 2.1.2. Artificial Digestion

The muscle samples were tested via artificial digestion (with the magnetic stirrer method) in the Parasitic Diseases Laboratory of FVM Timisoara. In total, 30 g of muscle was taken from the masseters and the tongue.

The method of artificial digestion was performed according to the EU Regulation 1478/2020 [13] in order to examine the samples of striated muscle tissue from the raccoon dog.

The main stages of artificial digestion were as follows:

The actual digestion of the muscle tissue and the release of the larvae consisted of the immersion of the sample in digestive fluid (hydrochloric acid and pepsin), which was followed by the continuous homogenization of the meat sample under continuous stirring (with a magnetic stirrer) at a temperature of 46 °C for 60 min until the muscle fragments were no longer visible.

The isolation of the larvae via sedimentation involved filtering the digestion fluid, after which it was left to rest for 30 min. The filtrate was then placed in a graduated test tube (40 mL), which was left to rest for 10 min. For the examination, 10 mL of the filtrate was retained.

The highlighting and counting of the larvae were performed using a stereo microscope. The *Trichinella* larvae were stored in 96% ethanol for further analysis of their molecular biology.

#### 2.1.3. DNA Extraction and Molecular Analysis

The molecular analysis was carried out at the “Victor Babeș” University of Medicine and Pharmacy in Timisoara. 

DNA extraction:

Following isolation, the pooled larvae were stored in ~1.5 mL 96% ethanol and kept at −20 °C for further use. Before DNA extraction, the samples were thawed and centrifuged (10 s, 15,000× *g*), and the supernatant was removed using a filtered micropipette tip without disturbing the larvae-containing sediment. The tubes were then left open to dry for approximately 45 min until no residual ethanol could be observed. 

Genomic DNA isolation was performed using a QIAamp Fast DNA Tissue Kit (Qiagen, Hilden, Germany), which included a ready-to-use proteinase K reagent, and the samples were eluted in 50 μL ATE buffer. The DNA concentration was verified spectrophotometrically using a Thermo Scientific™ NanoDrop (Thermo Fisher Scientific, Waltham, MA, USA). DNA was also extracted from reference larvae (supplied by RLT buffer) under the same conditions. The isolated DNA was stored at −20 °C until further use for the downstream PCR reactions.

Amplification of isolated DNA (multiplex PCR):

Genomic DNA from each genotype was amplified via multiplex polymerase chain reaction (PCR) including five primer pairs. The primer mixture was generated by combining equal volumes of the 10 primers, as described in [17]. The reference DNA (supplied by RLT buffer) was included as the positive control, and multi-Q-grade water was used as the negative control. The PCR reaction mix was prepared in 200 μL PCR tubes, which were then centrifuged at max speed (10 s). A total of 10 μL of the larval DNA to be tested was added to each tube, which was followed by another 10 s of centrifugation at max speed. All amplification reactions were carried out in an Applied Biosystems Veriti™ 96-Well Fast Thermal Cycler (Thermo Fisher Scientific, Waltham, MA, USA) under the conditions described in [17]. The tubes were then refrigerated at −20 °C preceding gel electrophoresis.

Gel electrophoresis:

The reaction products were mixed (1:5) with GelPilot DNA Loading Dye, 5x (Qiagen, Hilden, Germany), and subsequently separated on a 2% agarose gel followed by detection with ethidium bromide staining. Gel electrophoresis was performed using a horizontal electrophoresis chamber (Bio-Rad Laboratories, Hercules, CA, USA). For an easy and accurate sizing of the DNA fragments, 50 bp GelPilot DNA Molecular Weight Markers (Qiagen, Hilden, Germany) were loaded onto the 2% agarose gel (7 μL). The voltage was set at 90 V for the migration, and the gel was run for 45 min and then visualized under UV transillumination using Infinity Capture software (Version 6.5). 

DNA purification from the agarose gel:

Two fragments belonging to *T. britovi* were excised from the agarose gel. The first fragment had a total length of 129 bp and belonged to the ESV locus (primer sequences: forward, 5′-GTT.CCA.TGT.GAA.CAG.CAG.T-3′, and reverse, 5′-CGA.AAA.CAT.ACG.ACA.ACT.GC-3′). The second fragment had a total length of 253 bp and belonged to the ITS1 locus (primer 1 sequence: forward, 5′-GCT.ACA.TCC.TTT.TGA.TCT.GTT-3′, and reverse, 5′-AGA.CAC.AAT.ATC.AAC.CAC.AGT.ACA-3′; primer 2 sequence: forward, 5′-GCG.GAA.GGA.TCA.TTA.TCG.TGT.A-3′, and reverse, 5′-TGG.ATT.ACA.AAG.AAA.ACC.ATC.ACT-3′). The PCR products were sequenced by the Macrogen Europe^®®^ Company (Amsterdam, The Netherlands) and compared with those available in the GenBank database using BLAST alignment.

## 3. Results

Through artificial digestion, *Trichinella* spp. larvae were isolated from the muscle tissue of the raccoon dog. The larval biomass was estimated to be 23 larvae per gram (LPG) of muscle tissue.

The agarose gel results can be seen in Figure S1. The PCR products from the same sample were loaded in duplicate, and *T. britovi* (129 bp and 253 bp) was identified by comparing it with the RLT buffer reference larvae, also loaded onto the gel.

In the BLAST search, our sample closely matched (93%) the GenBank *T. britovi* sequence (KU374885) from Israel. Sequencing showed that the *Trichinella* nematode detected in the raccoon dog sample was identical to *T. britovi* sequences (GenBank no. KU374885.1) (Figures S2 and S3).

*T. britovi* is the most widely distributed species within the sylvatic cycles of Europe, Asia, and northern and western Africa [8]. Compared to *T. spiralis*, this species presents a low infectivity for rats and moderate infectivity for swine, slow nurse cell development, and a low production of newborn larvae in vitro [7]. In zoonotic terms, *T. britovi* is the second most common species of *Trichinella* that can affect human health [8]. *T. spiralis* is less resistant to frost species, *T. britovi* can survive in frozen carrion for up to one year, and *T. nativa* larvae can survive for up to five years [7].

## 4. Discussion

An overview of the European continent reveals that wildlife represents the most important reservoir of *Trichinella*, with wild animals being the most important source of infection for the domestic pig, which, in turn, is the main source of infection for other animals (e.g., horses), especially humans [8].

In accordance with the current European Union (EU) legislation on the control of *Trichinella* spp. in meat, Member States must implement a monitoring program for susceptible host species [13]. This involves the control of not only pigs but also wildlife in the monitored region. Thus, information about the factors that can favor the circulation of *Trichinella* spp. in nature is fundamental (characteristics of the habitat and wild host) in assessing the risk for domestic animals [8]. In Europe, *T. spiralis* (isolated from wild boar) and *T. britovi* (isolated from wild carnivores) are the most prevalent species in wild and domestic animals. *T. nativa* and *T. pseudospiralis* play a secondary role as pathogens for domestic animals, considering that *T. nativa* exclusively affects wild carnivores from arctic and subarctic areas and *T. pseudospiralis* has been identified in a small number of wild carnivores and sporadically in domestic pigs and humans [19]. The expansion of the raccoon dog, a recognized reservoir for *Trichinella*, can become a cause for concern in those geographical areas where more *Trichinella* species are circulating among wildlife [7]. In Romania, *Trichinella* infection is present and widespread throughout the country, significantly affecting wild animals. Both *T. spiralis* and *T. britovi* are present [10]. Animals affected by this nematode include fox, golden jackal, mink, wolf, wild cat, lynx, stone marten, badger, ermine, ferret, bear, and wild boar. *T. britovi* is the most widespread species in the sylvatic cycle in Romania [10,20,21,22,23,24,25,26,27,28,29,30,31].

The results of the present study complete the list of sylvatic hosts, with the addition of a new species, the raccoon dog, and attest to the remarkable presence of the *T. britovi* species in Romania. Analyzing our results, we noted that a study carried out in Holland [6] for the first time reported the presence of the species *T. spiralis* in the raccoon dog, which, despite its small population, may play an important role in the epidemiology of this disease. In Poland, the larvae of *T.* spiralis [32,33] and larvae of *T. britovi* [34] were identified in the musculature of raccoon dogs. One year later, juice from the muscles of this host was subjected to proteomic analysis, and the presence of heat shock proteins and serine protease was revealed to be associated with *T. britovi* infection [35]. In Denmark, this wild animal species represents a negligible reservoir for *Trichinella* species [5], while in Austria, the raccoon dog appears to be an important host for the zoonotic agents *E. multilocularis*, *Alaria alata*, and, to a lesser extent, species of the genus *Trichinella* [36].

In contrast, the biomass of *Trichinella* circulating in Estonia, in the sylvatic cycle, is considerable in the raccoon dog and fox, the identified species being *T. britovi* and *T. nativa* [37,38,39,40]. The reservoirs and sources of infection for domestic pigs in Germany are wild boars, raccoons (*Procyon lotor*), and foxes. *T. britovi, T. spiralis*, and *T. pseudospiralis* isolated from raccoon dogs have been reported in Germany [41,42,43,44,45].

*T. britovi,* the most widespread species in wild carnivores in Europe, also affects wildlife in Asia and North and West Africa. *T. britovi* can also affect domestic pig populations and is the second species of *Trichinella* that can affect human health [8,46].

The fact that *T. britovi* was identified in Romania and in the raccoon dog, among other host species (fox, jackal, wolf, wild cat, lynx, badger, stone marten, ermine, bear, and wild boar), once again confirms the hypothesis that these species constitute an important vector of trichinellosis transmission in the sylvatic environment with direct effects on species of hunting interest, particularly wild boar and bear, with a risk of human [10].

*T. britovi* and, to a lesser extent, *T. nativa* and *T. spiralis* were identified in the musculature of wild animals (jackal, fox, marten, badger, wolf, lynx, raccoon dog) from Latvia [47]. In Lithuania and Estonia, raccoon dogs and red foxes are strong reservoirs for helminths, including zoonotic ones, with *Trichinella* spp. and *A. alata* being the most prevalent parasites in the raccoon dog [4,48]. The risk of human infection is not associated with the species of the host animal but with the probability of its infection. In Romania, raw or undercooked wild boar meat represents a real source of infection with *Trichinella* spp. larvae, even when they are found in low numbers [49].

An outbreak of human trichinellosis in Poland due to the consumption of wild boar sausages that were not examined for *Trichinella* spp. was described by Różycki et al., 2022 [50]. Serbian researchers described the first cases of human trichinellosis in the winter of 2015–2016 as a result of the consumption of wild boar meat infected with *T. britovi* [51]. In Portugal, human infection with *T. britovi* has been reported among hunters who consume uncontrolled and undercooked wild boar meat [52].

In China, 27 cases of human infection have been reported, resulting from the consumption of mutton, dog, bear, wild boar, and raccoon dog [53].

The presence of *T. britovi* in host canid species located in peripheral habitats in rural localities where traditional hunting activities are frequently practiced may represent a route of transmission of this zoonosis to species of wild fauna of hunting interest (wild boar) and, through this channel, to people [10,12,54].

Carnivore–carnivore transmission, influenced by hunting practices adopted in certain areas of the world, supports the high prevalence of trichinellosis reported in northwest Europe in foxes, wolves, dogs, mustelids, and raccoon dogs, with the highest infection rate in wolves [55]. In the Western Alps, Italy, Martínez-Carrasco et al., 2023, describe the role of the wolf, which is an apex predator and a good maintenance host for *Trichinella* infection, with a high parasite intensity (>11.74 LPG) [56]. In Romania, too, wolves are good maintenance hosts for *Trichinella* infection with a high parasite intensity (>11.3 LPG) [20]. The larval biomass found in the muscle tissue of the raccoon dog (23 LPG) is low compared with that observed in other studies in Europe: in the Netherlands, the forelimb musculature (89.3 LPG) and the tongue (32.4 LPG) [6], in Poland (54.88 LPG) [34], in Estonia (58.8 LPG) [39].

*T. britovi, T. spiralis, T. pseudospiralis,* and *T. nativa* were hosted by the raccoon dog in a large study, in which 627 wild animals (fox, raccoon, lynx, wolf, badger, bear) from the north and southwest of Finland were examined [57]. Eight years later, the same species of the nematode were identified in Finland (2483 animals examined), with the raccoon dog and the fox being considered the most formidable reservoir animals for *T. spiralis, T. britovi, T. nativa,* and *T. pseudospiralis* [58].

The cadavers of raccoon dogs infected with the larvae of three taxa, *T. britovi, T. nativa,* and *Trichinella T6*, were preserved under the snow carpet. Testing of the larval survival time and reproductive capacity index revealed that this environment favored the survival of *Trichinella* larvae in host muscles, increasing the likelihood of their transmission to other hosts [59]. Other epidemiological investigations carried out in Japan, specifically in the Hokkaido region, identified the species *T. britovi* and the taxon *Trichinella T9*, respectively, in the musculature of the raccoon dog [60,61].

The identification of the species *T. britovi* in a new host in Romania, the raccoon dog, undoubtedly underlines the fact that this parasite reserve is maintained in the sylvatic environment at a consistent level, and knowledge of these reservoir hosts is a permanent concern for specialists in the veterinary and hunting fields.

## 5. Conclusions

The identification of *T. britovi*, a species with zoonotic potential, in the racoon dog, a wild carnivore host species expanding in Europe, represents a threat to the health of domestic animals and humans, in whom the evolution of the disease can be fatal.

Taking into consideration the fact that the only zoonotic species which circulate in Romania are *T. spiralis* and *T. britovi*, the present study provides a warning about the risk of transmission through new host species.

Analyzing the data obtained from the literature, the present study reports on *T. britovi* infection with the raccoon dog in Romania for the first time.

## Data Availability

Not applicable.

## Supplementary Materials

The following supporting information can be downloaded at https://www.mdpi.com/article/10.3390/pathogens12091132/s1, Figure S1. Agarose gel electrophoresis of DNA fragments obtained via multiplex PCR on raccoon dog larvae. 1. *T. britovi* reference larva; 2. *T. nativa* reference larva; 3. *T. pseudospiralis* reference larva; 4. *T. spiralis* reference larva; 5. *T. britovi reference larva*; 6. *T. nativa* reference larva; 7. *T. pseudospiralis* reference PCR products and 8. *T. spiralis* reference PCR products, supplied by RLT buffer; 11 and 12. larvae from the raccoon dog; Figure S2. Sequences of the ITS1 rDNA gene obtained from larvae isolated from raccoon dog; Figure S3. Sequences with the highest similarity to the PCR product obtained from the amplification of samples.
