# Supplementary material for: The First Identification of Trichinella britovi in the Raccoon Dog (Nyctereutes procyonoides) in Romania
Source: Pathogens. 2023 Sep 5;12(9):1132. doi: 10.3390/pathogens12091132 (PMC10535435; doi:10.3390/pathogens12091132)
Supplement: Supplementary file 1 [file pathogens-12-01132-s001.zip › pathogens-2558434-supplementary.pdf]

Supplementary Materials:

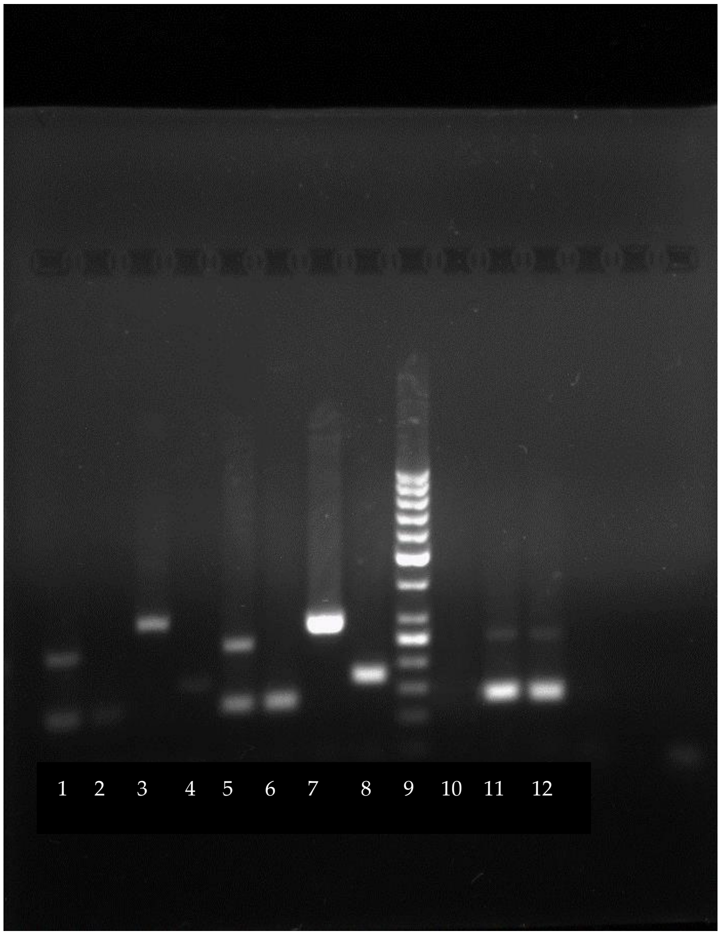

Figure S1. Agarose gel electrophoresis of DNA fragments obtained via multiplex PCR on raccoon dog larva. 1. *T. britovi* reference larva; 2. *T. nativa* reference larva; 3. *T. pseudospiralis* reference larva; 4. *T. spiralis* reference larva; 5. *T. britovi* reference larva; 6. *T. nativa* reference larva; 7. *T. pseudospiralis* reference PCR products and 8. *T. spiralis* reference PCR products, supplied by RLT; 11 and 12. larvae from the raccoon dog

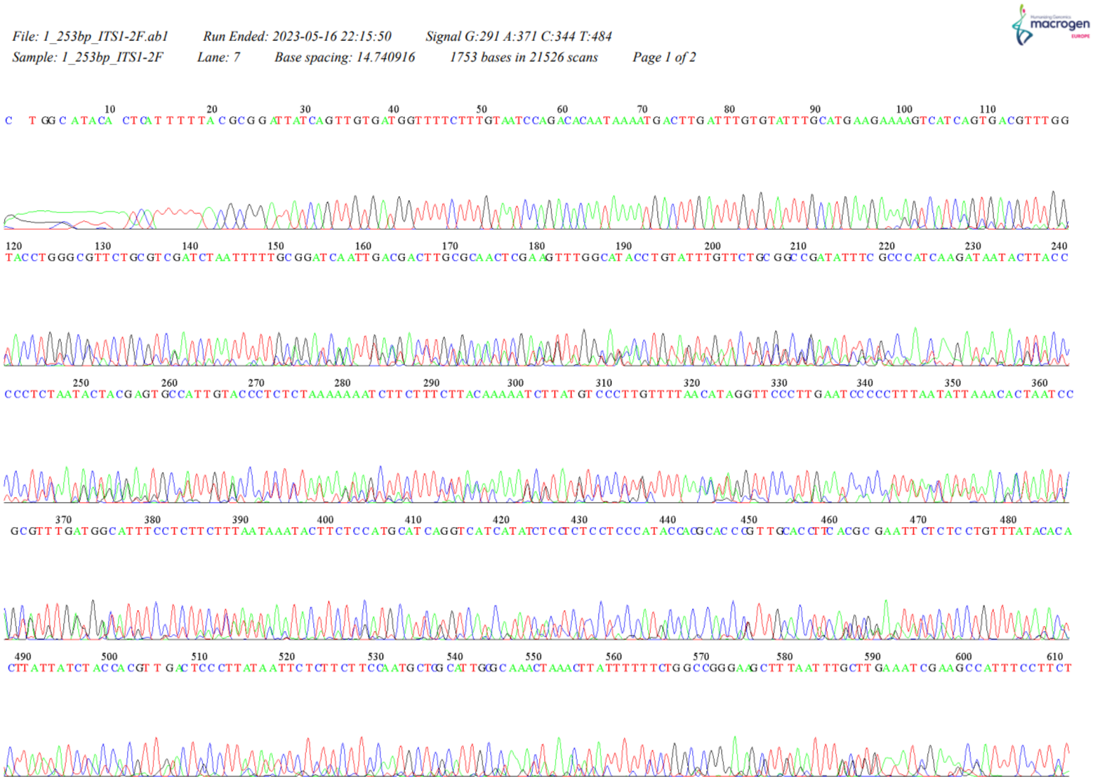

Figure S2. Sequences of the ITS1 rDNA gene obtained from larvae isolated from raccoon dog

GenBank ▾

Trichinella britovi isolate KVI_ITS_52_Tb_Vv 18S ribosomal RNA gene and internal transcribed spacer 1, partial sequence

GenBank: KU374885.1

FASTA Graphics PopSet

Go to:

```
LOCUS       KU374885                 547 bp    DNA     linear   INV 26-JUN-2016
DEFINITION  Trichinella britovi isolate KVI_ITS_52_Tb_Vv 18S ribosomal RNA gene
            and internal transcribed spacer 1, partial sequence.
ACCESSION   KU374885
VERSION     KU374885.1
KEYWORDS    .
SOURCE      Trichinella britovi
  ORGANISM  Trichinella britovi
            Eukaryota; Metazoa; Ecdysozoa; Nematoda; Enoplea; Dorylaimia;
            Trichinellida; Trichinellidae; Trichinella.
REFERENCE   1  (bases 1 to 547)
  AUTHORS   Erster,O., Roth,A., King,R. and Marcovics,A.
  TITLE     Direct Submission
  JOURNAL   Submitted (24-DEC-2015) Parasitology, Kimorn Veterinary Institute,
            POB 12, Bet Dagan, Israel 50250, Israel
COMMENT     ##Assembly-Data-START##
            Sequencing Technology :: Sanger dideoxy sequencing
            ##Assembly-Data-END##
FEATURES             Location/Qualifiers
     source          1..547
                     /organism="Trichinella britovi"
                     /mol_type="genomic DNA"
                     /isolate="KVI_ITS_52_Tb_Vv"
                     /host="Vulpes vulpes"
                     /db_xref="taxon:45882"
                     /country="Israel"
     misc_RNA        <1..>547
                     /note="contains 18S ribosomal RNA and internal transcribed
                     spacer 1"
ORIGIN
        1 ttcgcgccgg gaaatttcnc aaaccaaatc atntagagga agtaaaagtc gtaacaaggt
       61 ttccgtaggt gaacctgcgg aaggatcatt atcgtgtttt caaacgtaaa aagtgattgt
      121 tttgtgatgt gtgctaaaga gcaattgcac tttgttatgt gcagtgctga tgtgtgtatt
      181 tatcctcact gctgatgcgt gaaaatgatg ctacatcctt ttgatctgtg caaaaaggag
      241 attaataaat catttttaac gcggattatc agttgtgatg gttttctttg taatccagac
      301 acaataaaat gacttgattt gtgtatttgc atgaagaaaa gtcatcagtg acgtttggta
      361 cctgggcgtt ctgcgtcgat ctaatttttg cggatcgatt gacgacttgt gcaacatgac
      421 ggttggcagt agtgtagttg tactgtggtt gatattgtgt ctggctgtga tttgagttgt
      481 gttgcatttg ttgtgcagac gggtgcagtg gtttgtctat tttcatgtgt gtgatgtgtt
      541 gtgcatc
//
```

Figure S3. Sequences with the highest similarity to the PCR product obtained from the amplification of samples
